# Food Composition of the Diet in Relation to Changes in Waist Circumference Adjusted for Body Mass Index

**DOI:** 10.1371/journal.pone.0023384

**Published:** 2011-08-17

**Authors:** Dora Romaguera, Lars Ängquist, Huaidong Du, Marianne Uhre Jakobsen, Nita G. Forouhi, Jytte Halkjær, Edith J. M. Feskens, Daphne L. van der A, Giovanna Masala, Annika Steffen, Domenico Palli, Nicholas J. Wareham, Kim Overvad, Anne Tjønneland, Heiner Boeing, Elio Riboli, Thorkild I. Sørensen

**Affiliations:** 1 Department of Epidemiology and Biostatistics, School of Public Health, Imperial College London, United Kingdom; 2 Institute of Preventive Medicine, Copenhagen University Hospital, Copenhagen, Denmark; 3 National Institute for Public Health and the Environment (RIVM), Bilthoven, the Netherlands; 4 Department of Human Biology, Nutrition and Toxicology Research Institute of Maastricht (NUTRIM), Maastricht, the Netherlands; 5 Department of Clinical Epidemiology, Aarhus University Hospital, Aalborg, Denmark; 6 MRC Epidemiology Unit, Institute of Metabolic Science, Addenbrooke′s Hospital, Cambridge, United Kingdom; 7 Danish Cancer Society, Institute of Cancer Epidemiology, Copenhagen, Denmark; 8 Division of Human Nutrition, Wageningen University, Wageningen, the Netherlands; 9 Molecular and Nutritional Epidemiology Unit, ISPO (Cancer Research and Prevention Institute), Florence, Italy; 10 Department of Epidemiology, German Institute of Human Nutrition, Potsdam-Rehbruecke, Nuthetal, Germany; 11 Department of Epidemiology, School of Public Health, Aarhus University, Aarhus, Denmark; 12 Department of Cardiology, Aalborg Hospital, Aarhus University Hospital, Aalborg, Denmark; University of Las Palmas de Gran Canaria, Spain

## Abstract

**Background:**

Dietary factors such as low energy density and low glycemic index were associated with a lower gain in abdominal adiposity. A better understanding of which food groups/items contribute to these associations is necessary.

**Objective:**

To ascertain the association of food groups/items consumption on prospective annual changes in “waist circumference for a given BMI” (WC_BMI_), a proxy for abdominal adiposity.

**Design:**

We analyzed data from 48,631 men and women from 5 countries participating in the European Prospective Investigation into Cancer and Nutrition (EPIC) study. Anthropometric measurements were obtained at baseline and after a median follow-up time of 5.5 years. WC_BMI_ was defined as the residuals of waist circumference regressed on BMI, and annual change in WC_BMI_ (ΔWC_BMI_, cm/y) was defined as the difference between residuals at follow-up and baseline, divided by follow-up time. The association between food groups/items and ΔWC_BMI_ was modelled using centre-specific adjusted linear regression, and random-effects meta-analyses to obtain pooled estimates.

**Results:**

Higher fruit and dairy products consumption was associated with a lower gain in WC_BMI_ whereas the consumption of white bread, processed meat, margarine, and soft drinks was positively associated with ΔWC_BMI_. When these six food groups/items were analyzed in combination using a summary score, those in the highest quartile of the score – indicating a more favourable dietary pattern –showed a ΔWC_BMI_ of −0.11 (95% CI −0.09 to −0.14) cm/y compared to those in the lowest quartile.

**Conclusion:**

A dietary pattern high in fruit and dairy and low in white bread, processed meat, margarine, and soft drinks may help to prevent abdominal fat accumulation.

## Introduction

Body mass index (BMI) is commonly used in epidemiological studies as a measure of general adiposity, whereas waist circumference (WC) is an indicator of central adiposity and body fat distribution. Both measures have been associated with the risk of chronic disease morbidity and mortality; however, when WC is adjusted for BMI, this measurement is more strongly related to disease risk than BMI or WC alone, suggesting that this marker may capture the specific effect of the abdominal fat mass [1], [2], [3]. Changes in WC have been also positively associated with all-cause mortality in healthy middle-aged men and women after accounting for concurrent changes in BMI [4].

Several intervention studies have shown that there is a preferential mobilization of the abdominal fat, as compared to subcutaneous or total fat, among obese subjects in weight loss diets or other weight loss interventions [5], [6]. Hence, if we consider BMI-adjusted WC (WC_BMI_) to be a proxy measure of abdominal adiposity [7], then it might be possible that this measure is more sensitive to the effect of dietary factors compared to other measures of general adiposity such as body weight or BMI. In previous observational prospective studies conducted among European adults we have observed that several nutrients were associated with changes of WC and WC_BMI_; however these nutrients were not equally associated with changes in weight [8], [9], [10], [11], [12]. Specifically, we observed that a diet with a high glycemic index (GI) and high energy density (ED) was significantly associated with an increase in WC_BMI_. Given these results, we could speculate that low GI and low ED diets may prevent abdominal fat deposition.

In order to better interpret the effect of foods and diets on abdominal fat deposition, we aimed in the present study at assessing the association between specific food groups/items consumption and changes in WC_BMI_, i.e. changes in WC that are independent of concurrent changes in BMI. In addition, we assessed the effect of replacing some food groups/items for others, and the effect of combining several food groups/items on changes in WC_BMI_.

## Methods

### Participants

The current study included participants from eight centres in five countries involved in the European Prospective Investigation into Cancer and Nutrition (EPIC) study, participating in the DiOGenes (Diet, Obesity and Genes) project, namely Florence (Italy), Norfolk (UK), Amsterdam, Maastricht and Doetinchem (the Netherlands), Potsdam (Germany), Copenhagen and Aarhus (Denmark). Approval for this study was obtained from the ethical review boards of the International Agency for Research on Cancer (IARC) and from all local institutions where subjects had been recruited for the EPIC study: the Florence Health Authority Ethical Committee (Italy); the Norfolk Local Research Ethics Committee (UK); the Medical Ethics Committee of the Netherlands Organization for Applied Scientific Research (the Netherlands); the Ethics Committee of the Medical Association of the State of Brandenburg (Germany); and the Danish National Committee on Biomedical Research Ethics (Denmark). Written informed consent was obtained from all participants before joining EPIC study. Detailed information on the study population and data collection of the EPIC study has been described elsewhere [13].

At baseline (between1992–1998), participants filled out extensive questionnaires covering their diet, lifestyle, and medical history, and anthropometric measurements were obtained. Updated information on anthropometric data has been obtained from EPIC participants through follow-up examinations during 1998–2005 (median follow-up time 5.5 years). Of the 146,543 participants at baseline, 102,346 participated in the follow-up examination (69.8% response rate). Given that the current study forms part of larger project aiming at looking at gene*diet interactions in the development of abdominal adiposity, we excluded individuals with no blood samples collected (n = 4,048). We also excluded pregnant women (n = 133), those with missing information on diet or anthropometrics (n = 1,266), those in the lowest and highest 1% of the EPIC cohort distribution of the ratio of reported total energy intake: energy requirement (n = 752), individuals with prevalent chronic diseases (cancer, diabetes and/or cardiovascular disease) at baseline (n = 3,811) and incident chronic diseases during follow-up (n = 5,132), and those with unrealistic anthropometric measurements (n = 115). In order to avoid the variable changes in body composition and shape in old age and the possible underlying subclinical disease processes that occur with age, we excluded from the analyses participants with age at baseline >60 years or age at follow-up >65 years (n = 31,645). Finally, given the recognized effect of smoking and changes in smoking status on body weight and waist gain, those with missing information on smoking or changing smoking status between baseline and follow-up (n = 7,163) were excluded. In total 48,631 participants (19,694 men and 28,937 women) were included in the analyses (5,081 from Italy; 6,266 from the UK; 6,477 from the Netherlands; 8,661 from Germany; and 22,146 from Denmark).

### Dietary assessment

Usual food intakes were measured using country-specific validated food frequency questionnaires [14]. Estimated individual nutrient intakes, including total energy and alcohol, were derived from foods included in the dietary questionnaires through the standardized EPIC Nutrient Data Base (ENDB) [15].

The exposures of interest in this study were the consumption of food groups and specific food items in grams/day as well as the energy provided by these food groups/items (more information on the specific food groups/items in Table 1 and in Table S1).

**Table 1:** Average consumption of food groups/items (g/d) by gender in participants of the EPIC-DiOGenes project (n = 48,631).

	Men		Women	
	Mean (SD)	Range^1^	Mean (SD)	Range^1^
Potatoes	136.35 (84.18)	36.55–162.64	94.17 (65.00)	27.18–121.28
Vegetables	164.86 (91.39)	117.69–226.22	182.17 (102.35)	126.28–261.68
Legumes	5.54 (10.54)	0.82–17.25	5.28 (8.71)	1.33–14.92
Fruits	163.63 (135.89)	139.37–298.02	215.45 (150.76)	168.66–310.00
Nuts	4.19 (9.15)	0.95–13.57	2.77 (6.39)	0.72–9.06
Cereal products	256.77 (114.78)	194.89–465.88	205.35 (95.33)	184.16–306.32
Pasta and Rice	63.76 (72.37)	18.36–252.84	52.37 (58.61)	17.57–141.48
White Bread	38.17 (55.63)	23.22–158.92	30.29 (50.08)	13.49–110.74
Non-white Bread	123.29 (83.15)	23.36–172.58	91.96 (65.29)	25.64–123.21
Breakfast cereals^2^	15.09 (36.61)	1.67–45.95	14.67 (37.21)	2.11–52.41
Dairy products	367.98 (289.14)	229.59–470.98	347.68 (252.61)	260.85–422.42
Milk	238.44 (263.06)	80.20–364.15	200.45 (221.19)	81.30–332.93
Yogurt	56.76 (81.90)	29.73–66.34	74.81 (91.63)	43.65–91.72
Cheese	36.23 (29.04)	17.30–65.24	37.21 (28.32)	15.71–61.64
Meat products	146.96 (63.78)	163.22–99.77	98.91 (45.09)	91.44–106.05
Red meat	74.96 (43.11)	37.23–95.43	48.85 (30.04)	22.92–62.00
Poultry	21.91 (18.86)	11.65–30.96	19.13 (17.48)	10.24–28.21
Processed meat	46.73 (39.61)	30.02–95.26	28.47 (26.03)	19.80–58.34
Fish	35.55 (27.09)	9.61–45.82	30.09 (23.56)	10.23–38.44
Eggs	21.38 (17.99)	12.85–26.10	18.21 (15.58)	11.40–22.36
Fats & Oils	32.23 (17.02)	25.20–39.50	24.47 (14.57)	19.21–34.06
Vegetable oils	5.41 (8.99)	2.74–35.03	6.95 (11.03)	2.39–30.48
Butter	5.04 (10.20)	1.86–10.48	3.96 (8.02)	1.98–8.12
Margarine	20.12 (16.84)	0.34–25.60	12.66 (13.10)	0.32–16.13
Sugar & Confectionary	76.20 (97.09)	37.42–100.81	49.28 (48.70)	32.16–63.08
Cakes & Biscuits	39.83 (47.70)	22.35–79.50	36.74 (41.42)	18.46–57.77
Non-alcoholic beverages	1475.69 (688.49)	214.37–1764.78	1488.38 (836.36)	211.95–2050.52
Juices	63.76 (117.91)	31.19–189.97	76.50 (128.63)	35.24–199.77
Soft drinks	959.76 (501.82)	139.59–1138.79	863.22 (525.00)	154.84–1122.60
Coffee	682.07 (473.14)	108.26–886.03	522.77 (425.36)	111.74–733.10
Tea	258.72 (360.75)	29.25–615.13	300.36 (389.60)	40.66–584.03
Alcoholic beverages	408.19 (447.58)	273.72–487.47	137.33 (178.16)	73.60–183.91
Wine	92.24 (130.38)	28.73–215.83	77.30 (109.92)	38.98–95.67
Beer	305.52 (424.73)	46.08–379.28	53.59 (124.06)	17.48–81.17
Spirits	6.99 (15.53)	3.95–10.40	2.92 (8.55)	0.93–4.54

1 The range of exposure was expressed as the minimum and maximum mean consumption observed among the participating centres (Italy-Florence; UK-Norfolk; Netherlands-Amsterdam/Maastricht; Netherlands-Doetinchem; Germany-Potsdam; Denmark-Copenhagen/Aarhus).

2 These calculations were computed after excluding Italy where this food group was not assessed in their FFQ.

### Anthropometric measurements

At baseline, all participants were measured for weight, height and waist circumference. The methods used have been previously described in detail [16]. In brief, body weight and height were measured when participants wore light clothes and no shoes. Waist circumference was measured either at the midway between the lowest rib and the iliac crest (the Netherlands, and Potsdam-Germany) or at the narrowest torso circumference (the other centres). At follow-up examinations, participants in Norfolk (United Kingdom) and Doetinchem (the Netherlands) were measured by trained technicians using the same protocols as at baseline, whereas other centres provided self-reported data. For the latter, guidance was provided to measure waist circumference as at baseline, except for Denmark in which participants were guided to measure their waist circumference at the umbilicus (the reason for changing the site of measurement was to simplify the measurement instructions for participants).

The validity of the self-reported WC has been assessed in study carried out in 408 men and women from the Danish cohort. A high correlation between the self-reported and technician measured WC was found, but there was some underreporting and rather wide limits of agreement in the comparison. Miss-reporting of WC was associated with baseline BMI (men) and WC (women). It was, however, concluded that the self-reported WC could be used as a proxy for the technician-measured WC in regression analyses of changes in WC if these were adjusted for baseline BMI and WC [17].

Owing to the differences in the methods used to collect anthropometric data at follow-up and the length of follow-up, participants from Doetinchem (the Netherlands) were treated separately from those from Amsterdam and Maastricht (the Netherlands), whereas participants from Copenhagen and Aarhus (Denmark) were combined because no such differences between these two groups existed.

The outcome of interest in the present study was annual change in the phenotype WC_BMI_ (ΔWC_BMI_ in cm/year). This phenotype was defined both at baseline (baseline WC_BMI_) and at follow-up (follow-up WC_BMI_) as the residual values from the gender- and centre-specific regression equations of WC on BMI (using baseline and follow-up WC and BMI values respectively) [18], [19]. Annual changes in this phenotype (ΔWC_BMI_) were calculated as (follow-up WC_BMI_ – baseline WC_BMI_) / follow-up time. This method was used in our previous study [12].

### Assessment of other covariates

Information on age, gender, physical activity, educational level, smoking (never, former, and current smoker), menopausal status (premenopausal, peri-menopausal, and postmenopausal status), and use of hormone replacement therapy (yes/no or unknown) was collected through self-administered questionnaires at baseline. Physical activity level was indexed into five categories (inactive, moderately inactive, moderately active, active or unknown) based on occupational and recreational activities [20]. Educational level was inquired as the highest level of school achieved and participants were classified into primary school and less, technical–professional school, secondary school, university degree, or unknown.

### Statistical analyses

The association between dietary variables and ΔWC_BMI_ (in cm/year) was modelled using multi-adjusted linear regression analyses: centre-stratified analyses were carried out first, and random-effect meta-analyses were used to evaluate heterogeneity (*I* ^2^) across study centres, and to obtain pooled estimates of the associations.

The exposures of interest (consumption of food groups/items) were entered in the model as continuous variable per 100 kcal increments in intake, except for coffee and tea consumption, that were entered in the model per 100 g increments due to their very low energy content.

All analyses were adjusted for total energy intake (kcal), baseline age (years), baseline weight (kg), baseline height (cm), baseline WC_BMI_ (cm), smoking, alcohol intake (except for the model assessing the effect of alcoholic drinks on WC_BMI_ change), physical activity, education, and follow-up duration (years). In women, analyses were also adjusted for menopausal status and hormone replacement therapy use.

Analyses were conducted in men and women separately and in both genders combined. Plausible effect modification by gender in the association between food consumption and ΔWC_BMI_ was assessed by modelling an interaction term between gender and the exposure of interest; the effect modification by gender was considered significant when the p for interaction was <0.01 in ≥3 out of the 6 study centres.

For the food groups/items for which we found an association with ΔWC_BMI_, we constructed several models to ascertain the effect of substituting one food by one other, as previously described [21]. For that, the two food groups/items of interest were included simultaneously in the multiple adjusted models (per 100 kcal increments). The difference in the coefficients was used to estimate the effect of substituting 100 kcal of one food by 100 kcal of another.

Finally, in order to summarize the combined effect of the food groups/items that were significantly associated with ΔWC_BMI_ we constructed as summary score; for that we divided the consumption of these foods in sex-specific tertiles. A score value of 0, 1, or 2 was given to individuals within the first, second and third tertile of consumption of the foods that were inversely associated with ΔWC_BMI_; the scoring was reversed for the foods that were positively associated with ΔWC_BMI_. Thus, individuals with higher scores were more likely to present a dietary pattern that would prevent gains in WC_BMI_. The global association of this dietary pattern with ΔWC_BMI_ was assessed by comparing those with low scores (lower quartile) to those with higher scores (second, third and forth quartile).

It is important to note, both for the substitution models and for the summary scores, that the *posthoc* nature of these tests (since they are selected, based on initial results, among the full set of plausible models) makes it hard to directly interpret their evidential values in the statistical significance sense (e.g. through p-values and confidence intervals). Hence such information may primarily be seen as being of summarizing nature and, in fact, serving as a kind of upper bound for these summary findings.

All statistical analyses were performed with the STATA statistical package 10.0 (College Station TX).

## Results

The crude mean (SD) consumption and the range of exposure of food groups/items (in g/d) examined in the present study are shown in **Table 1**. More information on sex- and centre-specific means (SD) can be found in Table S1.

The consumption of several food groups/items was significantly associated with ΔWC_BMI_ in both men and women (**Table 2**). Fruit and dairy product consumption was inversely associated with gains in WC_BMI_; an increment of fruit consumption of 100 kcal was associated with −0.04 (95% CI −0.05 to −0.03) cm/y lower gain in WC_BMI_. The ΔWC_BMI_ corresponding to 100 kcal increment in dairy products consumption was −0.01 (95% CI −0.02 to −0.01) cm/y. On the other hand, consumption of white bread, processed meat, margarine, and soft drinks were all positively associated with ΔWC_BMI_: ΔWC_BMI_ corresponding to 100 kcal increment in consumptions of these food items were: 0.01 (95% CI 0.01 to 0.02) cm/y for white bread, 0.04 (95% CI 0.02 to 0.06) cm/y for processed meat; 0.03 (95% CI 0.01 to 0.05) cm/y for margarine; and 0.04 (95% CI 0.02 to 0.07) cm/y for soft drinks. Several other food groups/items were significantly associated with ΔWC_BMI_ in women only and in men and women combined: vegetables (negative association), potatoes, sugar & confectionary, beer and spirits (positive association). Finally, some foods were significantly associated with changes in WC_BMI_ only among women: vegetable oils, tea (negative association), and red meat (positive association). No significant interaction by gender was detected except for beer.

**Table 2:** Estimated association between food groups consumption^1^ and annual change in “waist circumference for a given body mass index (ΔWC_BMI_, cm/y)”.

		All			Men			Women	
	β^2^	(95% CI)	*P*	β^2^	(95% CI)	*P*	β^2^	(95% CI)	*P*
Potatoes	0.04	(0.01 to 0.06)*	*0.013*	0.02	(−0.01 to 0.05)	*0.120*	0.05	(0.01 to 0.09)	*0.014*
Vegetables	−0.08	(−0.11 to −0.03)	*<0.001*	−0.01	(−0.05 to 0.04)	*0.687*	−0.11	(−0.17 to −0.04)	*0.001*
Legumes	−0.03	(−0.17 to 0.12)	*0.705*	0.06	(−0.07 to 0.19)	*0.361*	−0.11	(−0.29 to 0.07)	*0.247*
Fruits	−0.04	(−0.05 to −0.03)	*<0.001*	−0.03	(−0.05 to −0.02)	*<0.001*	−0.05	(−0.07 to −0.03)	*<0.001*
Nuts	0.00	(−0.01 to 0.02)	*0.809*	0.00	(−0.02 to 0.03)	*0.702*	−0.00	(−0.04 to 0.03)	*0.834*
Cereal products	−0.00	(−0.01 to 0.01)	*0.944*	−0.00	(−0.01 to 0.01)*	*0.666*	0.01	(−0.00 to 0.01)	*0.097*
Pasta and Rice	−0.02	(−0.05 to 0.01)*	*0.228*	−0.02	(−0.05 to 0.00)	*0.086*	−0.01	(−0.05 to 0.03)*	*0.537*
White Bread	0.01	(0.01 to 0.02)	*<0.001*	0.01	(0.00 to 0.02)	*0.014*	0.01	(0.01 to 0.02)	*0.001*
Non-white Bread	−0.00	(−0.01 to 0.00)	*0.086*	−0.01	(−0.01 to 0.01)	*0.368*	−0.00	(−0.01 to 0.01)	*0.535*
Breakfast cereals^3^	−0.02	(−0.04 to −0.00)	*0.031*	−0.01	(−0.03 to 0.01)	*0.190*	−0.02	(−0.05 to 0.00)	*0.088*
Dairy products	−0.01	(−0.02 to −0.01)	*<0.001*	−0.01	(−0.02 to −0.01)	*<0.001*	−0.01	(−0.02 to −0.01)	*<0.001*
Milk	−0.01	(−0.02 to −0.00)	*0.005*	−0.01	(−0.02 to −0.00)	*0.013*	−0.02	(−0.03 to −0.01)	*0.005*
Yogurt	−0.02	(−0.03 to −0.01)	*<0.001*	−0.03	(−0.053 to −0.01)	*0.001*	−0.02	(−0.04 to −0.01)	*0.002*
Cheese	−0.01	(−0.02 to −0.00)	*0.007*	−0.01	(−0.02 to 0.00)	*0.082*	−0.01	(−0.02 to 0.00)	*0.073*
Meat products	0.02	(0.00 to 0.03)*	*0.036*	0.02	(−0.00 to 0.03)*	*0.117*	0.02	(0.01 to 0.04)*	*0.011*
Red meat	0.01	(−0.01 to 0.04)*	*0.207*	0.00	(−0.03 to 0.03)*	*0.946*	0.03	(0.01 to 0.05)	*0.004*
Poultry	−0.02	(−0.05 to 0.02)	*0.373*	0.01	(−0.04 to 0.04)	*0.815*	−0.02	(−0.06 to 0.02)	*0.269*
Processed meat	0.04	(0.02 to 0.06)*	*0.001*	0.02	(0.01 to 0.04)	*0.001*	0.05	(0.02 to 0.09)*	*0.004*
Fish	−0.05	(−0.10 to 0.00)*	*0.051*	−0.04	(−0.09 to 0.01)*	*0.137*	−0.05	(−0.01 to 0.00)*	*0.066*
Eggs	0.01	(−0.02 to 0.04)	*0.670*	−0.00	(−0.04 to 0.04)	*0.942*	0.01	(−0.03 to 0.05)	*0.747*
Fats & Oils	0.01	(−0.01 to 0.03)*	*0.167*	0.01	(−0.01 to 0.02)	*0.234*	0.02	(−0.01 to 0.04)*	*0.210*
Vegetable oils	−0.04	(−0.09 to 0.00)*	*0.062*	−0.04	(−0.10 to 0.03)*	*0.292*	−0.05	(−0.09 to −0.01)*	*0.020*
Butter	−0.01	(−0.02 to 0.01)	*0.451*	−0.00	(−0.02 to 0.01)	*0.570*	−0.01	(−0.03 to 0.01)	*0.494*
Margarine	0.03	(0.01 to 0.05)*	*0.001*	0.02	(0.00 to 0.04)	*0.037*	0.03	(0.02 to 0.05)	*<0.001*
Sugar & Confectionary	0.01	(0.00 to 0.01)	*0.013*	0.00	(−0.01 to 0.01)	*0.666*	0.01	(0.00 to 0.02)	*0.004*
Cakes & Biscuits	0.00	(−0.01 to 0.01)*	*0.909*	0.00	(−0.01 to 0.01)	*0.579*	−0.00	(−0.02 to 0.01)*	*0.704*
Non-alcoholic beverages	0.01	(−0.01 to 0.03)	*0.168*	0.01	(−0.01 to 0.02)	*0.322*	0.02	(−0.01 to 0.04)*	*0.252*
Juices	−0.01	(−0.03 to 0.00)	*0.100*	−0.01	(−0.02 to 0.01)	*0.315*	−0.02	(−0.05 to 0.01)	*0.211*
Soft drinks	0.04	(0.02 to 0.07)*	*0.002*	0.02	(0.00 to 0.04)	*0.018*	0.05	(0.02 to 0.09)	*0.007*
Coffee	0.00	(−0.00 to 0.00)	*1.000*	0.00	(−0.00 to 0.01)	*0.139*	−0.00	(−0.01 to 0.00)	*0.394*
Tea	−0.00	(−0.01 to 0.00)*	*0.110*	−0.00	(−0.01 to 0.01)	*0.848*	−0.01	(−0.01 to −0.00)	*0.018*
Alcoholic beverages	0.01	(0.00 to 0.02)	*0.001*	0.01	(−0.00 to 0.01)	*0.108*	0.02	(0.01 to 0.03)	*<0.001*
Wine	0.00	(−0.01 to 0.01)	*0.919*	−0.00	(−0.01 to 0.01)	*0.852*	0.00	(−0.02 to 0.03)*	*0.916*
Beer^4^	0.01	(0.00 to 0.02)	*0.041*	0.01	(−0.00 to 0.02)	*0.064*	0.05	(0.02 to 0.08)	*0.002*
Spirits	0.04	(0.01 to 0.06)	*0.003*	0.01	(−0.01 to 0.04)	*0.320*	0.13	(0.04 to 0.22)*	*0.007*

1 The effect of foods was studied per 100 kcal increments in intake, except for coffee and tea that were studied per 100 g increments due to their very low energy content.

2 The association between food consumption and ΔWC_BMI_ was modelled using centre-specific linear regression [adjusting for: total energy intake, age, baseline weight, baseline height, baseline WC_BMI_, smoking, alcohol intake (except in the models including alcoholic beverages), physical activity, education, follow-up duration, menopausal status (women only), and hormone replacement therapy use (women only)], and random-effect meta-analyses to evaluate heterogeneity (*I* ^2^) across study centres and to obtain pooled estimates of the associations.

*indicates that there is heterogeneity across study centres (*P* for heterogeneity <0.05).

3 After excluding Italy where this food group was not assessed in their FFQ.

4 Effect modification by gender was considered relevant when the p for interaction between the exposure of interest and gender was <0.01 in ≥3 out of the 6 study centres. Only Beer consumption met this criterion.

In order to get a more detailed picture of the association of the food composition of the diet on changes in WC_BMI_ we constructed several substitution models and summary scores. For that, we only considered the food groups/items that were consistently associated with ΔWC_BMI_, i.e. significant associations observed in both men and women, and no evidence of effect modification by gender. These analyses were conducted in the whole sample (men and women combined). Six food groups/items met these criteria: fruit, dairy product, white bread, processed meat, margarine, and soft drinks.

We analysed the change in the estimate of ΔWC_BMI_ observed when fruits or dairy products replaced the consumption of white bread, processed meat, margarine, and soft drinks in an iso-caloric diet (**Table 3**). Regarding dairy products, the largest change in WC_BMI_ was observed when dairy products substituted soft-drinks: hence, in an iso-caloric diet, substituting 100 kcal of dairy for100 kcal of soft-drinks was associated with a −0.05 (95% CI −0.07 to −0.03) cm/y increase in WC_BMI_. The largest change in WC_BMI_ observed when fruit consumption replaced the consumption of other food items corresponded also to soft-drinks: when 100 kcal of fruit replaced 100 kcal of soft-drink, the corresponding decrease in WC_BMI_ was −0.08 cm (95% CI −0.09 to −0.06).

**Table 3:** Estimated annual change in “waist circumference for a given body mass index (ΔWC_BMI_, cm/y)” observed when 100 kcal of fruits or 100 kcal of dairy products replaced 100 kcal of white bread, processed meat, margarine, or soft drinks (analyses conducted in men and women combined).

		All
	β^1^	(95% CI)
Dairy products replacing^2^:		
White bread	−0.02	(−0.03 to −0.02)
Processed meat	−0.04	(−0.07 to −0.03)
Margarine	−0.03	(−0.04 to −0.03)
Soft drinks	−0.05	(−0.07 to −0.03)
Fruits replacing^3^:		
White bread	−0.05	(−0.06 to −0.05)
Processed meat	−0.07	(−0.09 to −0.06)
Margarine	−0.06	(−0.06 to −0.06)
Soft drinks	−0.08	(−0.09 to −0.06)

1 The association between food consumption and ΔWC_BMI_ was modelled using centre-specific linear regression [adjusting for: total energy intake, age, baseline weight, baseline height, baseline WC_BMI_, smoking, alcohol intake, physical activity, education, follow-up duration, menopausal status (women only), and hormone replacement therapy use (women only)], and random-effect meta-analyses to evaluate heterogeneity (*I* ^2^) across study centres and to obtain pooled estimates of the associations.

2 Four models were constructed, all of them including dairy product consumption (per 100 kcal increments) plus either white bread (model 1), processed meat (model 2), margarine (model 3), or soft drinks (model 4), also in 100 kcal increments.

3 Four models were constructed, all of them including fruit consumption (per 100 kcal increments) plus either white bread (model 1), processed meat (model 2), margarine (model 3), or soft drinks (model 4), also in 100 kcal increments.

To estimate the global association between a diet that simultaneously combined these six food groups/items and ΔWC_BMI_, we created a summary score of our main results: participants within the 1^st^, 2^nd^, and 3^rd^ sex-specific tertile of fruit and dairy products consumption were given 0, 1, and 2 points respectively; participants within the 1^st^, 2^nd^, and 3^rd^ sex-specific tertile of white bread, processed meat, margarine, and soft drinks were given 2, 1, and 0 points respectively. The points given to these six food groups/items were summed, so the overall score range was 0–12 points. The mean (SD) score in the whole sample was 6.00 (2.20). In descending order, the mean (SD) score in each study centre was: 7.11 (1.74) in Italy; 6.35 (2.16) in the UK; 6.07 (2.26) in Denmark; 5.59 (2.06) in the NL-Doetinchem; 5.36 (1.98) in Germany; and 5.28 (2.24) in the NL-Amsterdam/Maastricht (data not shown in tables). The score was divided into four categories according to the distribution in the sample (quartiles): 0–4 points (25.29% of the sample); 5–6 (33.41%); 7–8 (28.05%); and 9–12 (13.24%). Therefore, participants in the highest category were likely to have a diet rich in fruit and dairy products and low in white bread, processed meat, margarine, and soft drinks.

**Figure 1:**
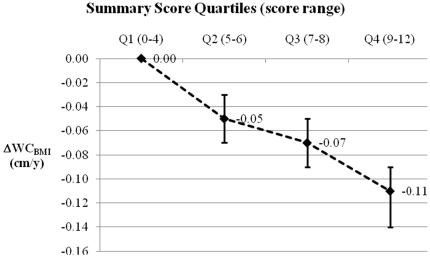
Estimated global association between a summary score reflecting a dietary pattern with a high content of fruit and dairy products, and low content of white bread, processed meat, margarine, and soft drinks and annual change in “waist circumference for a given body mass index (ΔWC_BMI_, cm/y)”. The association between the quartiles of the summary score (quartile 1 or Q1 is the reference category) and ΔWC_BMI_ was modelled using centre-specific linear regression [adjusting for: total energy intake, age, baseline weight, baseline height, baseline WC_BMI_, smoking, alcohol intake, physical activity, education, follow-up duration, menopausal status (women only), and hormone replacement therapy use (women only)], and random-effect meta-analyses to obtain pooled estimates of the associations.

Compared to the first category of the score, those in the second, third, and forth category showed a ΔWC_BMI_ of −0.05 (95% CI −0.03 to −0.07), −0.07 (95% CI −0.05 to −0.09), and −0.11 (95% CI −0.09 to −0.14) cm/y respectively (**Figure 1**). There was no evidence of heterogeneity by study centre (Figure S1).

## Discussion

In the present study we have observed that among European men and women, a dietary pattern characterized by a high consumption of fruits and dairy products, and a low consumption of white bread, processed meat, margarine, and soft drinks was associated with a lower gain in WC_BMI_, i.e. gains in WC that are independent of concurrent gains in BMI and are likely to represent abdominal fat accumulation. Stronger associations were detected when fruits and dairy products replaced the consumption of white bread, processed meat, margarine, and soft drinks in an iso-caloric diet, or when these food items were analyzed in combination using a single summary score. Other food groups/items were also significantly associated with ΔWC_BMI_ among women (vegetables, potatoes, sugar & confectionary, beer and spirits, vegetable oils, tea, and red meat).

Strengths of this study include its prospective design; the inclusion of a large sample size of adults from different European countries with diversity in their dietary intakes; the application of a strict exclusion criteria so as to eliminate from the sample participants who may have changed their weight or WC as a results of other factors potentially confounding the effects of diet, but difficult to adjust for (i.e. older individuals, those with prevalent and incident chronic diseases, those with unknown smoking status or changes in their smoking status); and the possibility to control for a large number of plausible confounders.

Some limitations should also be considered: the assessment of diet using food frequency questionnaires and the use of self-reported anthropometry are subjected to measurement error. We could not explore the association of specific food groups with ΔWC_BMI_, such as low-fat dairy products, given that this information was not available. Misreporting of diet is a major concern in epidemiological studies looking at the association between diet and measurements of body fatness; however, in prospective studies it is less likely that weight-dependent bias in reporting the diet influence the prospective change in body composition, when baseline weight and waist are controlled for. Different centres used different techniques for WC measurement. In order to deal with this, each centre was treated as a different cohort, and centre-specific associations were estimated and pooled using random effect meta-analysis in order to capture these differences. Indeed, despite of these differences between centres in their anthropometrical measurements, we observed that all centres showed almost identical effect estimates and hence no evidence of heterogeneity between centres was detected. Finally, anthropometric values were self-reported at follow-up in 4 out of the 6 study centres, and this may have introduced some bias due to the selective under-reporting of weight and/or WC among the overweight or the obese. A validation study was conducted in a sub-sample of the Danish cohort to estimate the agreement between the measured and self-reported anthropometric values. Results of this validation study showed a very high correlation between the self-reported and technician measured WC and weight, but there was some degree of miss-reporting associated to both baseline BMI and WC. However, it was concluded that the self-reported measurements could be used a proxy of technician-measured in regression analyses if these were adjusted for baseline BMI and WC [17]. Therefore all our models have been adjusted for baseline measurements. In addition, a recent publication showed similar associations between baseline measured and follow-up self-reported WC and BMI and total mortality, indicating that self-reported anthropometry can predict morality risk as well as the measured values, and therefore self-reported anthropometry can be considered a good proxy measure of obesity for obesity and health association studies [4].

Previous epidemiological studies on this topic were either cross-sectional in nature, included small sample sizes or did not adjust WC changes for concurrent BMI changes; nevertheless, in agreement with the present results, previous research also reported an association between consumption of refined grains and abdominal adiposity [22], [23] or BMI-adjusted gains in WC [24]. Higher dairy products intake, dietary calcium and serum vitamin D have been also associated with lower gains in WC [25], weight [26], and with less intra-abdominal adipose tissue accumulation [27]. Similar to our results, a previous study using cluster analyses found that individuals with a dietary pattern characterized by a high consumption of “white bread” showed a greater waist gain compared to individuals within the “healthy pattern” (i.e. rich in fruit, vegetables, reduced-fat dairy, whole grains and low in red and processed meat, fast food, and soda) [28]. Similar associations have been reported among children and adolescents [29].

The dietary pattern associated to lower gain in abdominal obesity observed in this and previous studies, was similar to the Dietary Approaches to Stop Hypertension (DASH)-diet. This diet features high intakes of fruit, vegetables, legumes, and nuts; moderate amounts of low-fat dairy products; and low amounts of animal protein and sweets [30]. This diet was originally designed for blood pressure reduction [31], although it has also been shown to reduce low-density lipoprotein cholesterol levels [32] and the risk of developing chronic diseases such as coronary heart disease [33] or colorectal cancer [34]. An intervention study conducted among individuals with the metabolic syndrome showed that the DASH-diet reduced metabolic risk factors, including their WC after 6 months of intervention [35]. Among diabetic subjects, those allocated to a DASH-diet intervention also reduced significantly their WC more than those allocated to the control diet [36]. Despite a few differences between the dietary factors associated with lower abdominal fat gain in the present study and the DASH-diet (i.e. we did not detect an association between legumes and nuts consumption and ΔWC_BMI_), it seems plausible that dietary patterns similar to the DASH-diet exert additional effects on abdominal fat deposition, which may in part explain its protective effect against the incidence of chronic diseases associated with obesity.

In a previous study based on the present sample, GI and ED were the only nutritional factors significantly associated with ΔWC_BMI_ in both men and women; alcohol, glycemic load, and fibre also were associated with ΔWC_BMI_ among women only [12]. It is then likely that the observed effect of food groups/items significantly associated with changes in WC_BMI_ were mediated in part through these nutritional factors. As expected, fruit and dairy products consumption was significantly and inversely correlated with both ED and GI in the present sample, and white bread, margarine and soft drinks were positively correlated with ED and GI; processed meat consumption was positively correlated with ED and negatively correlated with GI (data not shown). Apart from these nutritional factors, it might be possible that the effect of these food groups/items on ΔWC_BMI_ is mediated through other nutrients that were not addressed in the previous study, such as calcium, vitamin D or *trans*-fatty acids.

In order to better translate these findings into public health messages that encourage overall healthy diets we constructed a summary score of our results. This summary score should be interpreted with caution given that it is neither a hypothesis oriented score, nor was empirically-derived *a posteriori* using data driven techniques: it was constructed to ascertain the overall combined effect of all the food groups/items associated with ΔWC_BMI_, and hence to take into account the multidimensionality of diet, correlations between food intakes, as well as plausible cumulative or synergistic effects of these foods on ΔWC_BMI_ (i.e. effect of a single food may not be detectable whereas the cumulative effects of multiple foods included in a food pattern may be sufficiently large to be detectable). Overall it was estimated that those following a diet characterized by high fruit and dairy products and low white bread, processed meat, margarine, and soft drinks consumption, would show 1.1 cm less gain in WC for a given gain in BMI during a 10 years period, compared to those with a diet with opposite characteristics; nevertheless, this estimate may have been underestimated due to the measurement error associated with the measurement of both diet and anthropometric changes.

We constructed a second summary score which additionally included potatoes, vegetables, sugary & confectionary, and spirits, as these food groups/items were shown to be associated with ΔWC_BMI_ in women and in the whole sample (score range 0-20); the association between this second summary score and ΔWC_BMI_ was similar to that observed using the simpler version of the score in the whole sample; additional effects of the second summary score on WC_BMI_ changes were detected among women only.

Finally, it was demonstrated that a more pronounced change of ΔWC_BMI_ could be obtained when the foods preventing abdominal fat gain in the diet replaced the consumption of foods promoting abdominal fat gain.

In conclusion, results from this study suggest that a food composition of the diet characterized by a high consumption of dairy products and fruits and a low consumption of white bread, processed meat, margarine, and soft drinks – similar to the DASH-diet – may help to prevent abdominal fat accumulation among European men and women. In addition, this study supports that a whole dietary pattern incorporating simultaneously several food recommendations may yield further benefits on health – in this case prevention of abdominal fat accumulation, compared to the effect of its single components, and hence recommendations should encourage an overall healthy diet. These results are in concordance with the previously reported protective effect of low GI and ED diets against abdominal fat accumulation.

## Supporting Information

Figure S1 Estimated centre-specific association between a summary score reflecting a dietary pattern with a high content of fruit and dairy products, and low content of white bread, processed meat, margarine, and soft drinks and annual change in “waist circumference for a given body mass index (ΔWC_BMI_, cm/y)”. The association between the quartiles of the summary score (quartile 1 or Q1 is the reference category) and ΔWC_BMI_ was modelled using centre-specific linear regression [adjusting for: total energy intake, age, baseline weight, baseline height, baseline WC_BMI_, smoking, alcohol intake, physical activity, education, follow-up duration, menopausal status (women only), and hormone replacement therapy use (women only)], and random-effect meta-analyses to obtain pooled estimates of the associations.
(TIF)

Table S1 Consumption of food groups/items (g/d) by gender and centre in participants of the EPIC-DiOGenes project (n = 48,631).
(DOC)
