# Supplementary figures and images for: Food Composition of the Diet in Relation to Changes in Waist Circumference Adjusted for Body Mass Index
Source: PLoS One. 2011 Aug 17;6(8):e23384. doi: 10.1371/journal.pone.0023384 (PMC3157378; doi:10.1371/journal.pone.0023384)

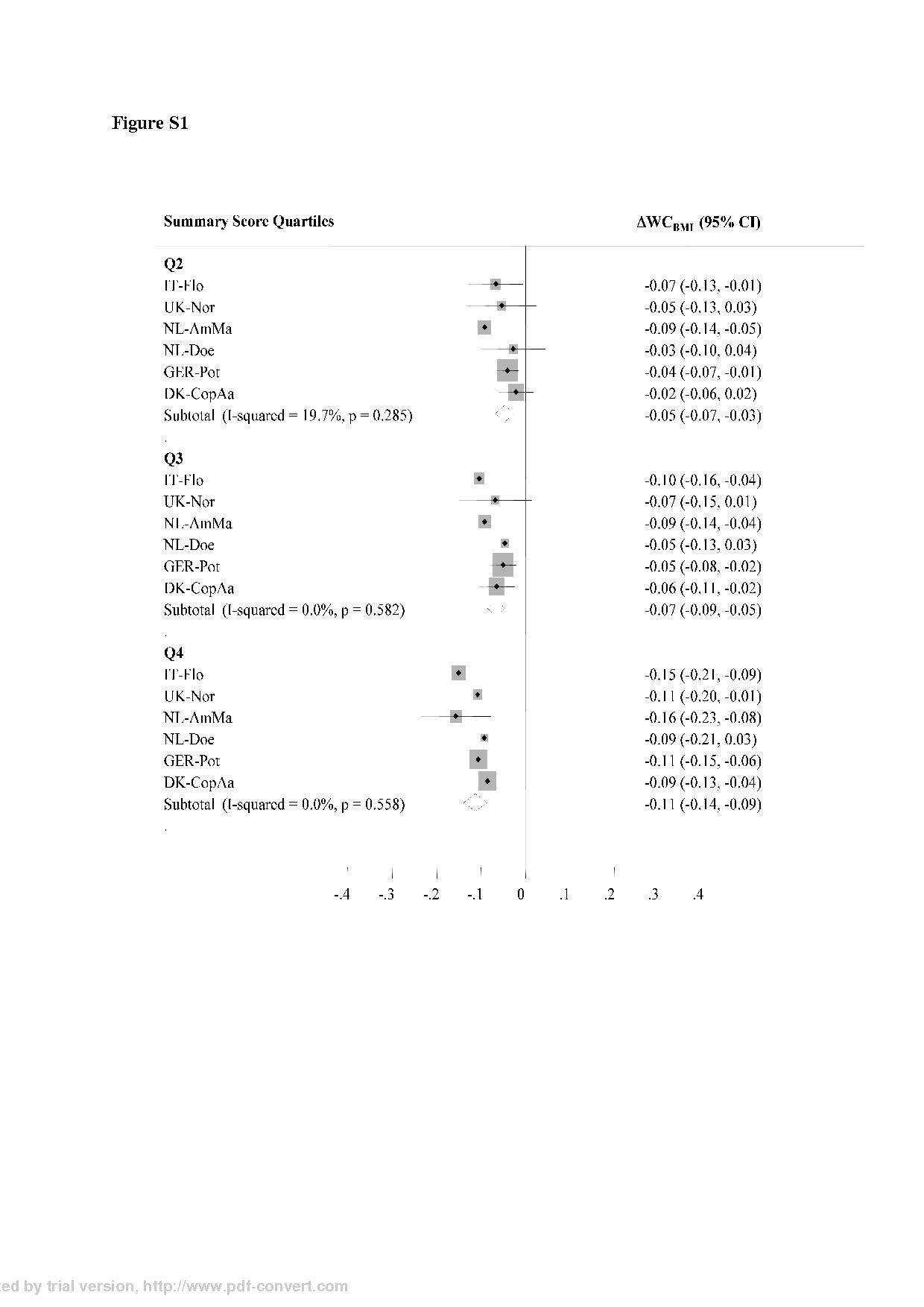

Supplement: Figure S1 — Estimated centre-specific association between a summary score reflecting a dietary pattern with a high content of fruit and dairy products, and low content of white bread, processed meat, margarine, and soft drinks and annual change in “waist circumference for a given body mass index (ΔWCBMI, cm/y)”. The association between the quartiles of the summary score (quartile 1 or Q1 is the reference category) and ΔWCBMI was modelled using centre-specific linear regression [adjusting for: total energy intake, age, baseline weight, baseline height, baseline WCBMI, smoking, alcohol intake, physical activity, education, follow-up duration, menopausal status (women only), and hormone replacement therapy use (women only)], and random-effect meta-analyses to obtain pooled estimates of the associations. (TIF) [file pone.0023384.s001.tif]
