# Supplementary material for: Food Composition of the Diet in Relation to Changes in Waist Circumference Adjusted for Body Mass Index
Source: PLoS One. 2011 Aug 17;6(8):e23384. doi: 10.1371/journal.pone.0023384 (PMC3157378; doi:10.1371/journal.pone.0023384)
Supplement: Table S1 — Consumption of food groups/items (g/d) by gender and centre in participants of the EPIC-DiOGenes project (n = 48,631). (DOC) [file pone.0023384.s002.doc]

Table S1: Consumption of food groups/items (g/d) by gender and centre in participants of the EPIC-DiOGenes project (n = 48,631)

|  |  | Men | | | | | | Women | | | | | |
| --- | --- | --- | --- | --- | --- | --- | --- | --- | --- | --- | --- | --- | --- |
|  |  | IT-  Flo | UK-  Nor | NL-  AmMa | NL-  Doe | GER-  Pot | DK-  CopAa | IT-  Flo | UK-  Nor | NL-  AmMa | NL-  Doe | GER-  Pot | DK-  CopAa |
| Potatoes | mean | 36.55 | 113.22 | 127.68 | 151.02 | 105.16 | 162.64 | 27.18 | 103.44 | 85.34 | 98.64 | 78.34 | 121.28 |
|  | *SD* | *30.70* | *62.66* | *78.85* | *73.96* | *52.55* | *89.43* | *22.85* | *58.16* | *53.73* | *54.43* | *44.02* | *69.04* |
| Vegetables | mean | 174.57 | 226.22 | 123.16 | 117.69 | 121.52 | 173.84 | 183.88 | 261.68 | 132.26 | 126.28 | 132.61 | 196.00 |
|  | *SD* | *96.90* | *109.69* | *48.88* | *43.53* | *58.72* | *91.37* | *101.57* | *121.20* | *53.04* | *43.77* | *62.35* | *103.12* |
| Legumes | mean | 11.46 | 17.25 | 10.15 | 10.48 | 4.05 | 0.82 | 10.04 | 14.92 | 8.08 | 9.13 | 2.19 | 1.33 |
|  | *SD* | *8.84* | *19.34* | *11.59* | *10.01* | *4.11* | *1.54* | *8.24* | *15.26* | *8.77* | *8.23* | *2.55* | *2.43* |
| Fruits | mean | 298.02 | 187.66 | 158.82 | 170.33 | 139.37 | 149.08 | 310.00 | 246.33 | 176.30 | 197.90 | 168.66 | 205.94 |
|  | *SD* | *175.47* | *140.64* | *131.15* | *125.45* | *94.47* | *132.62* | *168.00* | *169.02* | *125.50* | *122.15* | *99.89* | *150.55* |
| Nuts | mean | 0.95 | 3.83 | 13.45 | 13.57 | 3.52 | 2.13 | 0.72 | 3.25 | 8.67 | 9.06 | 2.55 | 1.62 |
|  | *SD* | *2.85* | *8.49* | *16.38* | *17.11* | *6.67* | *4.31* | *1.65* | *7.44* | *10.92* | *12.13* | *5.78* | *3.52* |
| Cereal products | mean | 465.88 | 194.89 | 287.85 | 257.07 | 245.31 | 247.87 | 306.32 | 184.16 | 208.06 | 193.56 | 182.24 | 190.73 |
|  | *SD* | *176.59* | *115.53* | *110.02* | *92.42* | *87.52* | *85.41* | *133.09* | *108.32* | *79.79* | *69.41* | *65.28* | *69.41* |
| Pasta and Rice | mean | 252.84 | 42.26 | 78.46 | 60.55 | 18.36 | 59.86 | 141.48 | 46.51 | 59.08 | 49.14 | 17.57 | 40.63 |
|  | *SD* | *130.36* | *44.57* | *64.76* | *48.85* | *16.00* | *44.03* | *91.62* | *42.53* | *49.33* | *42.01* | *15.21* | *29.80* |
| White Bread | mean | 158.92 | 44.08 | 42.63 | 32.36 | 23.22 | 27.50 | 110.74 | 26.31 | 23.94 | 18.96 | 13.49 | 15.70 |
|  | *SD* | *104.35* | *49.93* | *59.19* | *55.24* | *26.03* | *34.28* | *81.29* | *33.64* | *34.64* | *28.91* | *16.31* | *24.34* |
| Non-white Bread | mean | 26.36 | 49.13 | 140.26 | 139.83 | 172.58 | 133.96 | 25.64 | 47.67 | 98.79 | 100.63 | 123.21 | 110.00 |
|  | *SD* | *50.15* | *59.61* | *88.26* | *83.57* | *86.01* | *67.47* | *45.75* | *49.91* | *59.48* | *51.02* | *62.43* | *56.48* |
| Breakfast cereals^1^ | mean |  | 45.95 | 5.68 | 4.56 | 1.67 | 13.98 |  | 52.41 | 4.63 | 3.77 | 2.11 | 12.23 |
|  | *SD* |  | *79.62* | *15.52* | *12.60* | *6.16* | *20.46* |  | *79.90* | *11.08* | *8.57* | *5.80* | *18.79* |
| Dairy products | mean | 229.59 | 436.68 | 427.60 | 470.98 | 254.10 | 376.80 | 260.85 | 422.42 | 373.69 | 458.35 | 274.75 | 368.88 |
|  | *SD* | *176.59* | *190.31* | *328.57* | *306.00* | *237.80* | *309.45* | *181.86* | *183.94* | *271.08* | *259.60* | *218.13* | *281.96* |
| Milk | mean | 134.62 | 364.15 | 251.82 | 267.17 | 80.20 | 259.40 | 155.56 | 332.93 | 206.84 | 257.40 | 81.30 | 222.14 |
|  | *SD* | *157.16* | *176.27* | *270.73* | *258.62* | *159.80* | *289.46* | *154.93* | *165.82* | *216.50* | *216.61* | *136.60* | *254.21* |
| Yogurt | mean | 29.73 | 30.97 | 55.67 | 63.01 | 55.38 | 66.34 | 43.65 | 49.01 | 62.77 | 75.17 | 80.92 | 91.72 |
|  | *SD* | *56.42* | *44.71* | *80.74* | *73.83* | *74.80* | *92.44* | *68.19* | *59.83* | *88.25* | *82.84* | *98.42* | *99.54* |
| Cheese | mean | 65.24 | 17.30 | 41.49 | 41.60 | 41.26 | 34.80 | 61.64 | 15.71 | 36.67 | 36.75 | 35.90 | 36.49 |
|  | *SD* | *40.89* | *14.21* | *33.78* | *29.81* | *29.56* | *25.46* | *37.24* | *12.71* | *26.04* | *22.64* | *23.38* | *25.42* |
| Meat products | mean | 135.39 | 99.77 | 135.11 | 137.18 | 149.22 | 163.22 | 106.05 | 91.63 | 91.44 | 97.99 | 93.26 | 102.74 |
|  | *SD* | *63.96* | *51.27* | *64.19* | *52.88* | *70.74* | *58.56* | *49.21* | *49.16* | *49.18* | *43.12* | *46.35* | *40.35* |
| Red meat | mean | 66.95 | 42.11 | 73.61 | 80.84 | 37.23 | 95.43 | 52.83 | 39.87 | 53.99 | 62.00 | 22.92 | 59.70 |
|  | *SD* | *40.39* | *30.49* | *36.71* | *32.65* | *24.34* | *39.44* | *31.12* | *30.70* | *31.96* | *30.20* | *14.86* | *26.08* |
| Poultry | mean | 30.96 | 25.80 | 15.37 | 11.65 | 14.65 | 24.52 | 26.34 | 28.21 | 11.88 | 10.24 | 10.54 | 20.37 |
|  | *SD* | *22.98* | *19.18* | *15.22* | *9.67* | *12.84* | *19.74* | *18.84* | *21.41* | *11.29* | *9.48* | *9.81* | *17.36* |
| Processed meat | mean | 31.51 | 30.02 | 44.78 | 43.59 | 95.26 | 38.80 | 22.11 | 21.87 | 24.67 | 24.96 | 58.34 | 19.80 |
|  | *SD* | *24.68* | *23.95* | *38.31* | *31.49* | *56.36* | *25.77* | *17.27* | *17.27* | *22.45* | *18.19* | *35.59* | *14.07* |
| Fish | mean | 33.56 | 35.74 | 11.27 | 9.61 | 26.63 | 45.82 | 29.12 | 37.41 | 11.17 | 10.23 | 20.13 | 38.44 |
|  | *SD* | *22.67* | *26.27* | *10.27* | *8.68* | *25.15* | *25.99* | *21.12* | *26.65* | *11.60* | *9.94* | *20.94* | *22.01* |
| Eggs | mean | 16.42 | 12.85 | 17.40 | 17.75 | 18.84 | 26.10 | 17.30 | 11.40 | 14.48 | 15.58 | 16.32 | 22.36 |
|  | *SD* | *11.14* | *11.85* | *14.66* | *14.31* | *13.96* | *20.42* | *10.65* | *10.20* | *12.01* | *11.40* | *14.74* | *18.23* |
| Fats & Oils | mean | 39.50 | 28.20 | 37.18 | 39.02 | 36.64 | 29.40 | 34.06 | 22.93 | 26.15 | 26.27 | 29.04 | 19.21 |
|  | *SD* | *15.27* | *18.45* | *19.32* | *17.51* | *18.79* | *14.63* | *14.19* | *16.01* | *13.88* | *12.92* | *14.74* | *11.85* |
| Vegetable oils | mean | 35.03 | 5.19 | 4.31 | 2.74 | 4.10 | 3.03 | 30.48 | 3.89 | 4.46 | 2.86 | 4.33 | 2.39 |
|  | *SD* | *14.55* | *3.42* | *4.36* | *3.41* | *3.39* | *4.08* | *13.44* | *2.83* | *3.81* | *2.99* | *3.47* | *3.19* |
| Butter | mean | 1.86 | 4.61 | 6.53 | 5.48 | 10.48 | 3.56 | 1.98 | 3.89 | 4.84 | 3.64 | 8.12 | 2.61 |
|  | *SD* | *3.78* | *10.48* | *7.64* | *6.66* | *14.74* | *9.00* | *3.61* | *8.59* | *6.18* | *4.39* | *11.28* | *6.92* |
| Margarine | mean | 0.34 | 18.40 | 20.17 | 25.60 | 21.07 | 21.77 | 0.32 | 15.15 | 12.59 | 16.13 | 16.00 | 13.94 |
|  | *SD* | *0.81* | *17.54* | *16.12* | *15.00* | *17.89* | *16.06* | *0.64* | *15.13* | *10.71* | *10.55* | *14.20* | *12.39* |
| Sugar & Confectionary | mean | 46.12 | 57.63 | 59.33 | 63.08 | 37.42 | 100.81 | 39.42 | 45.38 | 43.78 | 44.20 | 32.16 | 63.08 |
|  | *SD* | *31.59* | *43.68* | *40.58* | *38.61* | *30.22* | *126.31* | *30.05* | *38.35* | *31.23* | *28.38* | *25.98* | *62.90* |
| Cakes & Biscuits | mean | 55.25 | 79.50 | 28.46 | 28.11 | 68.12 | 22.35 | 50.60 | 57.77 | 27.93 | 31.41 | 57.69 | 18.46 |
|  | *SD* | *51.10* | *70.82* | *25.10* | *23.89* | *61.71* | *22.55* | *47.22* | *53.40* | *21.52* | *22.02* | *52.75* | *18.29* |
| Non-alcoholic beverages | mean | 214.37 | 1250.04 | 1401.79 | 1366.06 | 1284.87 | 1764.78 | 211.95 | 1230.12 | 1421.42 | 1427.91 | 1372.02 | 2050.52 |
|  | *SD* | *166.64* | *414.56* | *551.80* | *459.41* | *595.32* | *641.60* | *151.08* | *426.90* | *545.17* | *479.16* | *632.50* | *682.99* |
| Juices | mean | 34.72 | 45.83 | 81.77 | 59.11 | 189.97 | 31.19 | 36.83 | 55.94 | 87.75 | 88.76 | 199.77 | 35.24 |
|  | *SD* | *63.71* | *62.75* | *111.13* | *86.28* | *215.30* | *52.41* | *70.81* | *70.22* | *95.46* | *98.73* | *212.07* | *57.81* |
| Soft drinks | mean | 139.59 | 1075.50 | 812.75 | 924.38 | 670.65 | 1138.79 | 154.84 | 1047.35 | 802.39 | 852.83 | 702.83 | 1122.60 |
|  | *SD* | *88.94* | *400.37* | *390.89* | *349.99* | *398.32* | *473.49* | *104.38* | *399.09* | *382.42* | *305.23* | *422.18* | *482.83* |
| Coffee | mean | 108.26 | 460.37 | 593.42 | 722.72 | 445.91 | 886.03 | 111.74 | 463.32 | 473.48 | 545.23 | 405.01 | 733.10 |
|  | *SD* | *71.03* | *359.74* | *388.34* | *368.52* | *331.98* | *470.59* | *73.59* | *360.17* | *349.11* | *289.68* | *282.71* | *463.43* |
| Tea | mean | 29.25 | 615.13 | 197.98 | 184.06 | 121.51 | 252.76 | 40.66 | 584.03 | 282.16 | 271.06 | 119.86 | 389.51 |
|  | *SD* | *64.93* | *375.30* | *238.79* | *205.14* | *231.78* | *376.91* | *83.13* | *369.30* | *286.11* | *242.42* | *219.56* | *453.11* |
| Alcoholic beverages | mean | 273.72 | 233.52 | 318.63 | 294.00 | 447.49 | 487.47 | 114.87 | 73.60 | 108.03 | 74.11 | 121.04 | 183.91 |
|  | *SD* | *247.47* | *325.11* | *395.66* | *388.56* | *423.63* | *491.30* | *155.55* | *118.08* | *156.75* | *122.23* | *164.79* | *201.50* |
| Wine | mean | 215.83 | 52.69 | 47.26 | 28.73 | 61.92 | 113.62 | 93.45 | 45.11 | 50.02 | 38.98 | 67.23 | 95.67 |
|  | *SD* | *214.28* | *86.04* | *87.07* | *66.35* | *92.15* | *135.14* | *135.71* | *72.52* | *84.47* | *66.57* | *95.29* | *118.98* |
| Beer | mean | 46.08 | 172.77 | 257.65 | 250.39 | 379.28 | 362.81 | 17.48 | 22.25 | 47.25 | 18.90 | 51.11 | 81.17 |
|  | *SD* | *81.90* | *299.64* | *385.21* | *386.90* | *418.60* | *465.79* | *51.28* | *84.75* | *122.01* | *89.45* | *121.55* | *147.08* |
| Spirits | mean | 6.69 | 5.08 | 10.06 | 10.40 | 3.95 | 7.51 | 1.67 | 2.56 | 4.54 | 4.05 | 0.93 | 3.94 |
|  | *SD* | *16.43* | *11.79* | *28.40* | *25.11* | *9.27* | *12.82* | *5.81* | *7.29* | *16.91* | *14.41* | *3.16* | *8.04* |

Abbreviations: IT-Flo (Italy-Florence); UK-Nor (UK-Norfolk); NL-AmMa (Netherlands-Amsterdam/Maastricht); NL-Doe (Netherlands-Doetinchem); GER-Pot (Germany-Potsdam); DK-CopAa (Denmark-Copenhagen/Aarhus)

^1^ Breakfast cereal consumption was not assessed in the Italian FFQ.
